# Choledocholithiasis in a Patient Presenting 12-Years Post-Cholecystectomy: A Case Report

**DOI:** 10.51894/001c.165685

**Published:** 2026-07-31

**Authors:** Bobbie Love, Mary Hughes, Mckenzie Farthing

**Affiliations:** 1 University of Michigan Health - Sparrow

## 95

### Introduction

Regardless of the time post cholecystectomy, the physician should maintain a high index of suspicion for choledocholithiasis. The percentage of post-cholecystectomy patients with common bile duct (CBD) stones ranges from 20.1% at 2 days to 2.2% at 6 weeks.1,2

### Case presentation

An 82-year-old male with extensive medical history including cholecystectomy in 2013, presented with a 3-day history of nausea, anorexia, hematuria, fatigue and pain with deep inhalation “under my (right) rib cage”. Vital signs were normal. Physical exam revealed a diaphoretic male with a soft abdomen and minimal RUQ tenderness. Differential included duodenitis, choledocholithiasis, cholangitis, sphincter of Odi dysfunction, ACS, and GERD. Initial lab results revealed transaminitis, direct hyperbilirubinemia, elevated alkaline phosphatase, leukocytosis and lactic acidosis. CT abdomen/pelvis w/contrast showed features of duodenitis, and the CBD measured 10mm (normal post-op). Gastroenterology was consulted, patient was provided fluids, acetaminophen, ondansetron, linezolid and piperacillin/tazobactam intravenously, and admitted. Magnetic resonance cholangiopancreatography (MRCP) noted two stones in the distal CBD. Endoscopic retrograde cholangiopancreatography (ERCP) revealed multiple ulcers of the duodenal bulb and three CBD stones ranging from 5 - 10 mm were removed. The patient was discharged on antibiotics and pantoprazole. His stent was successfully removed with repeat ERCP.

### Discussion

CBD stones must remain on the differential regardless of the length of the time since cholecystectomy. In choledocholithiasis, CT has a 65% sensitivity versus 96% for MRCP.3 The CT did not reveal abnormal findings for his post-operative history. The CBD stones were not visualized. The MRCP showed two stones, and three stones were removed by ERCP.

